# Impact of hearing loss on brain signal variability in older adults under different auditory load conditions

**DOI:** 10.3389/fnagi.2025.1498666

**Published:** 2025-02-27

**Authors:** Songjian Wang, Tong Liu, Yi Liu, Nuonan Kou, Younuo Chen, Yuan Wang, Wenjian Sun, Shuo Wang

**Affiliations:** ^1^Key Laboratory of Otolaryngology Head and Neck Surgery, Department of Otolaryngology-Head and Neck Surgery, Beijing Tongren Hospital, Ministry of Education, Beijing Institute of Otolaryngology, Capital Medical University, Beijing, China; ^2^College of Electronic Information Engineering, Yantai University, Yantai, China

**Keywords:** age-related hearing loss, speech-in-noise, speech perception, brain signal variability, neural resource allocation

## Abstract

**Introduction:**

The moment-by-moment variability in brain signals, a newly recognized indicator, demonstrates both the adaptability of an individual’s brain as a unique trait and the distribution of neural resources within that individual in response to constantly shifting task requirements. This study aimed to explore brain signal variability in older adults using oxyhemoglobin (HbO) variability derived from fNIRS during tasks with increasing signal-to-noise ratio (SNR) loads and to assess the effects of varying degrees of hearing loss on speech recognition performance and related brain signal variability patterns.

**Methods:**

Eighty-one participants were categorized into three groups: healthy controls (*n* = 30, aged 65.5 ± 3.4), mild hearing loss (*n* = 25, aged 66.0 ± 3.7), and moderate to severe hearing loss (*n* = 26, aged 67.5 ± 3.7). Speech perception was tested under quiet, 5 dB SNR, and 0 dB SNR conditions.

**Results:**

Results revealed that the brain signal variability increased with higher SNR loads in healthy older adults, indicating enhanced neural resource allocation with the SNR load. In contrast, we found that hearing loss reduced brain signal variability during speech recognition tasks, especially in noisy conditions, in the mild hearing loss and moderate to severe hearing loss groups, possibly indicating decreased neural processing efficiency. Additionally, a positive correlation between brain signal variability and speech recognition performance was observed in healthy control participants across all SNR conditions, suggesting that brain signal variability could dynamically respond to the precise level of auditory environment demands. However, this relationship was only significant at the 5 dB SNR condition in hearing loss groups.

**Discussion:**

Taken together, this study underscores the significant impact of hearing loss on brain signal variability modulation in auditory cognitive tasks and highlights the need for further research to understand the underlying neural mechanisms.

## Introduction

Aging is associated with a range of cognitive and sensory declines, including hearing loss, which significantly impacts the quality of life in older adults (Tremblay et al., 2021). Hearing loss is not merely a peripheral sensory deficit but also involves changes in central auditory processing and brain function. Specifically, hearing loss has been shown to significantly impact various cognitive functions, including memory, attention, and executive functioning (Slade et al., 2020). According to the sensory deprivation hypothesis (Humes et al., 2013), long-term hearing loss leads to the redistribution of cognitive resources to auditory perception over time, resulting in a decline in cognitive ability. This functional change may lead to alterations in brain signal variability, which is crucial for efficient neural processing (Li et al., 2024).

Brain signal variability reflects the dynamic adaptability and flexibility of neural systems, and greater variability has been linked to better cognitive performance in various tasks. In the field of cognitive neuroscience, most studies concentrate on deriving conclusions from average brain activation patterns, while the moment-to-moment brain signal variability is often considered as conceptualized “noise” (Garrett et al., 2010; Steinberg et al., 2022). However, recent neuroimaging research has demonstrated the variability of brain signal is a critical component of brain function, enabling the nervous system to adapt to constantly changing internal and external demands and make appropriate behavioral responses (Koen and Rugg, 2019; Li et al., 2024). This is complementary to the theory that biological variation is necessary for optimal brain function (Nomi et al., 2017). According to the coordination dynamics theory, the brain is a metastable configuration that dynamically fluctuates between integration and isolation. The variability of brain neural signals is the basis for high integration or segregation brain networks to flexibly transition between metastable configurations (Nomi et al., 2017; Tognoli and Kelso, 2014). Evidence has suggested that brain signal variability within individuals can dynamically adjust to specific levels of environmental demands (Garrett et al., 2014; Grundy et al., 2019; Li et al., 2024), and the potential neural mechanisms of intra-individual variability (Boylan et al., 2021). Specifically, linking brain signal variability with multiple indicators of task performance, such as accuracy and reaction time, could help understand individual differences in brain variability behind cognitive performance. Variability in brain signals may demonstrate utility as a novel measure of individual differences in cognitive neuroscience (Mohr and Nagel, 2010).

Previous research has predominantly focused on how cognitive load affects brain signal variability during tasks involving working memory and attention (Grundy et al., 2019; Steinberg et al., 2022). During the n-back working memory task, an increase in task difficulty is associated with greater variability in brain signals, which correlates with faster and more stable response times (Steinberg et al., 2022). Similarly, numerous studies have shown that variability in brain function may have practical applications as a new method for measuring individual differences in cognitive neuroscience (Good et al., 2020; McGinley et al., 2015; Rieck et al., 2022; Zhang et al., 2021). In addition, previous research has found that brain signal variability across large-scale brain regions may be impacted by the developmentally aging process (Rieck et al., 2022). Some perspectives maintain that older adults demonstrate reduced individual variability in brain responses when confronted with continuously changing cognitive demands. Specifically, compared to younger individuals, older adults exhibit less fluctuation in brain activity when transitioning from non-task-relevant regions to various task-relevant regions (Cieslak et al., 2018; Rieck et al., 2022). This limited variability in core cognitive control areas may contribute to the working memory deficits commonly observed in the aging process. These studies suggest that increased brain signal variability provides the flexibility to shift between different cognitive states, enhancing the dynamic range and contributing to more effective cognitive performance on the tasks being performed. However, opposite findings is also reported that older adults show greater brain signal variability across primarily cortical regions during the fixation period of a task, compared with younger individuals (Boylan et al., 2021; Garrett et al., 2013). It is plausible that brain signal variability may exhibit both increases and decreases across the adult lifespan, with the effects of these fluctuations potentially differing across various brain regions (Boylan et al., 2021). Prior studies conducted by our lab and other research groups have investigated that age-related hearing loss (characterized by gradually developing high-frequency hearing loss) is often accompanied by declines in auditory function (Du et al., 2016; Wang et al., 2024; Yang et al., 2024). Prior neuroimaging research has revealed an increased activation in prefrontal regions associated in adults with age-related hearing loss. This enhanced activation is believed to reflect a compensatory strategy employed to improve auditory performance (Proskovec et al., 2016). However, the impact of auditory cognitive load, particularly in the context of hearing loss, on brain signal variability remains underexplored. Understanding these dynamics is essential, as they may reveal compensatory mechanisms or deficiencies in neural processing associated with hearing loss.

The main objective of this study was to utilize HbO variability derived from the fNIRS technique to explore how brain signal variability in older adults changes in response to tasks with increasing signal-to-noise ratio (SNR) loads. Additionally, we aimed to examine the effects of varying degrees of hearing loss on speech recognition performance and related brain signal variability patterns. By doing so, we seek to provide a deeper understanding of the neural mechanisms underlying auditory processing in older adults with hearing loss and to identify potential biomarkers for assessing and managing auditory cognitive decline. In this study, we hypothesize that brain signal variability will increase with lower SNR loads in healthy older adults, reflecting greater neural resource allocation and cognitive flexibility. Conversely, we expect that hearing loss will reduce brain signal variability, particularly under noisy conditions, indicating a less adaptive neural system. Furthermore, we anticipate that greater brain signal variability will be associated with better speech recognition performance, but this relationship may be disrupted in individuals with hearing loss.

## 2 Materials and methods

### 2.1 Sample

Eighty-four subjects were recruited and participated in the study. Participants were excluded from participating if they reported a history of neurological disorders, prior use of hearing aids or cochlear implants, and cognitive-function-affecting drug use or treatment. The general cognitive function of each subject was examined using the Mini-Mental State Examination (MMSE) and Montreal Cognitive Assessment (MoCA), while depression symptoms were evaluated using the Geriatric Depression Scale (GDS) and University of California at Los Angeles Loneliness Scale (UCLA). Each participant fully understood the purpose of the study and provided written informed consent before the experiment. All patients recruited in this study were able to independently follow the study instructions. Due to participants’ incorrect execution of task procedures or low data quality, three participants were excluded from all data analysis. Experiment was approved by the local ethics committee (Institutional Review Board of Beijing Institute of Otolaryngology and Beijing Tongren Hospital), and was performed in accordance with the Declaration of Helsinki.

### 2.2 Audiometry

Hearing loss was assessed using the speech-frequency pure tone average (PTA), measured with a clinical audiometer at frequencies of 0.25 kHz, 0.5 kHz, 1 kHz, 2 kHz, 4 kHz, and 8 kHz. Based on PTA results, thirty participants were categorized into the healthy control (HC) group (PTA < 25 dB HL at 0.25 kHz–4 kHz bilaterally, 14.6 ± 3.4 dB HL, Range: 8.8 dB HL–20.0 dB HL), 25 participants were categorized into the mild hearing loss (M_HI) group (PTA 26–40 dB HL at 0.25 kHz–4 kHz bilaterally, 33.1 ± 3.9 dB HL, Range: 26.8 dB HL–35.3 dB HL), and 26 participants were categorized into the moderate to severe hearing loss (MS_HI) group (PTA > 40 dB HL at 0.25 kHz–4 kHz bilaterally, 46.6 dB HL ± 3.4 dB HL, Range: 41.8 dB HL–63.8 dB HL), using a Melison audiometer following WHO standards (Chadha et al., 2021). The audiogram can be found in the Supplementary Figure 1.

### 2.3 Speech perception test

**FIGURE 1:**
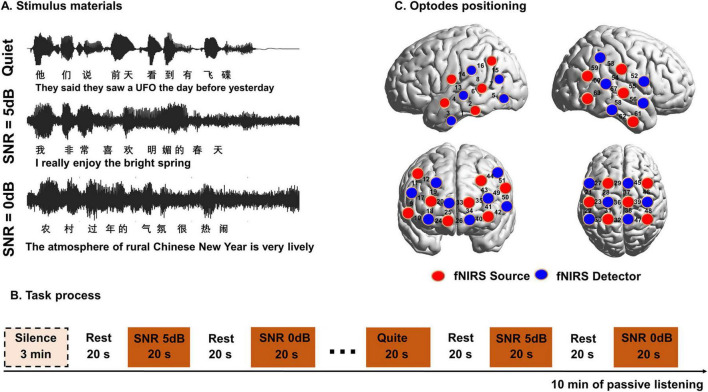
The speech stimuli and fNIRS task process. **(A)** Speech stimuli for the experimental assessments. Sample waveform was presented to illustrate the sound conditions in a quiet environment, signal-to-noise ratio (SNR) of 5 dB, 0 dB. **(B)** The pseudorandom block design of stimulation. **(C)** Placement of the channel of fNIRS. The red buttons represent the fNIRS emitting diode sources, the blue buttons represent the fNIRS detectors.

The speech perception stimuli material consisted of 12 easily understood sentence lists, each containing 20 sentences with 10 words per sentence spoken by a male talker. These lists were obtained from the Mandarin Hearing Test in Noise (MHINT) (Wong et al., 2007), for example, “I really enjoy the bright spring.” Three test conditions were adjusted to two levels of SNR (5 dB and 0 dB) using speech-shaped noise (SSN) and the quiet condition (Figure 1A). The SNR quantified the level difference between the speech signal and the background white noise, with 5 dB SNR indicating the speech was 5 dB louder than the noise and 0 dB SNR indicating equal levels. Sentences with varying SNR conditions and in quiet were randomly presented to participants, with each SNR condition repeated five times. Participants were asked to repeat as many words as they could recognize at the end of each sentence and were encouraged to guess if they were not certain under each condition. The speech intensity was 65 dB, delivered through a speaker positioned 1 m directly in front of the subject, with testing conducted in an isolation chamber where background noise levels were ≤ 30 dB. Participants received instructions and practice before the experiment. Speech recognition scores for each participant were then obtained by calculating the number of correct words in each sentence under various conditions.

### 2.4 fNIRS data acquisition

The assessment of brain performance during speech perception was carried out using a block-designed fNIRS task, similar to the approach utilized in our previous studies. The acoustic stimuli material utilized in the fNIRS experiment were also derived from MHINT software akin to those used in behavioral experiments. Each fNIRS session started with 3 min of resting in silence, and three conditions of acoustic stimuli (quiet, SNR 5 dB, and SNR 0 dB) were presented in a block design, with 20 s sound presentation blocks interleaved with silence blocks of 20 s (Figure 1B). Each stimulus condition was randomly presented across five blocks to ensure that no condition repeated consecutively. The experimental procedure was programmed based on the Psychotoolbox 3.0 extensions in MATLAB 2020b (MathWorks).

fNIRS data was acquired using a NirScan-9000A device equipped with 24 light-emitting diode sources and 24 avalanche photodiode detectors positioned on the temporal, parietal, and frontal areas of the scalp. Each source-detector pair, spaced 3 cm apart, constituted a channel, resulting in a total of 63 channels. Measurements of oxyhemoglobin (HbO) and deoxyhemoglobin (HbR) concentrations in the cerebral cortex were obtained using near-infrared light at wavelengths of 730 nm, 808 nm, and 850 nm, sampled at 11 Hz. A three-dimensional (3D) digital locator (Patriot, Polhemus, United States) was employed for optode positioning, referencing the nasal root point, central point, left preauricular point, and right pre-auricular point. These coordinates were then registered to the Montreal Neurological Institute and Hospital (MNI) standard brain template using spatial registration in NirSpace (Danyang Huichuang Medical Equipment Co., Ltd., China). The regions of interest (ROI) for scanning included areas pertinent to the dual route models of human speech perception (Friederici et al., 2017) and prefrontal regions associated with related to auditory function compensation (Du et al., 2016), encompassing the left superior temporal gyrus (STG), left middle temporal gyrus (MTG), Broca’s area, Wernicke’s area, left dorsolateral prefrontal cortex (DLPFC), left ventral premotor cortex (PMv), and corresponding regions in the right hemisphere, as illustrated in Figure 1C and the detailed positioning can be found in Supplementary Table 1.

### 2.5 Data pre-processing

The FC-NIRS software package was used to evaluate the quality of the fNIRS data. Channels were considered invalid and subsequently excluded from further analysis if the heart rate (1 Hz∼1.5 Hz) was not detectable or if the signal-to-noise ratio (SNR) fell below 10 (Xu et al., 2015). Ultimately, three subjects were identified with unusable data and were consequently excluded from further analysis. The valid recordings were then preprocessed using the NIRS_KIT toolbox (Hou et al., 2015), including (1) the conversion of optical density according to the Modified Beer-Lambert law (MBLL), (2) motion artifact correction based on the method called temporal derivative distribution repair (TDDR) (Fishburn et al., 2019) which effectively removes baseline shift and spike artifacts, (3) detrending using the wavelet-MDL to exclude physiological noise such as heart rate, and breathing (Yang et al., 2024), and (4) bandpass filtering (0.01 Hz–0.2 Hz). For the current study, only HbO data was analyzed because previous studies have shown that HbO changes are more sensitive than HbR in determining cerebral blood flow changes. Additionally, HbO data exhibit a higher signal-to-noise ratio (Li et al., 2024). The mean of the 5 s before the onset of the block was chosen as the baseline for baseline correction and an additional 10 s after the block’s end was included to allow sufficient time for the hemodynamic response function (HRF) to return to baseline. This process resulted in a total block-averaged time series of 35 s.

### 2.6 HbO variability calculation

After preprocessing the fNIRS data, intra-individual brain signal variability in HbO was quantified by calculating the standard deviation (SD) of the HbO time series for the channel of interest. Compared to alternative metrics (such as mean squared successive differences), the SD maintains the same scale as the original time series, rendering it a more appropriate measure for evaluating the temporal variability of short time series, such as those found in block design tasks (Steinberg and King, 2024; Zhang et al., 2018). Each SNR condition included 1,925 data points (35 s task duration × 5 repetitions × sampling rate of 11 Hz). Significantly, to minimize the differences in signal magnitude, we applied z-score normalization to standardize the HbO signal for each channel before computing the SD HbO. This variability assessment is crucial for understanding neural adaptation to dynamic environments and stability maintenance in diverse scenarios (Garrett et al., 2013; Halliday et al., 2018; Li et al., 2024).

### 2.7 Behavioral partial least squares analysis

Behavioral Partial Least Squares (PLS) analysis was a multivariate statistical, data-driven technique to identify significant relationships between brain activity and task performance. The PLS method in neuroscientific research explored meaningful structures by modeling channel covariance and avoided multiple comparison corrections (Krishnan et al., 2011; Meidenbauer et al., 2021). In addition, the data-driven nature reduces the impact of individual researcher biases (Romero et al., 2015). We employed the behavioral PLS to investigate the relationships between HbO variability and speech perception performance by accessing inter-individual effects in three groups. The goal of this analysis was to explore whether higher levels of SD HbO associate with better speech perception performance at each SNR load, and to identify the main control brain regions that maintain this association.

In the present study, behavioral PLS was performed to investigate the correlation between performance and channel-specific HbO variability across three SNR levels in three groups. The behavioral data consisted of averaged speech recognition score for each SNR level for each participant during the speech perception behavioral test and the fNIRS brain data consisted of the SD of the HbO corresponding to changes in HbO time series for each SNR level for each participant. Specifically, behavioral PLS relies on singular value decomposition (SVD) applied to a covariance matrix. For example, consider the healthy control group, matrix X represents HbO variability, and matrix Y represents speech recognition score, both of which are mean-centered and normalized. Matrix X (90 × 63) consist of 30 subjects across 63 channels, with three matrices corresponding to each SNR level. Matrix Y (90 × 1) comprises three 30 × 1 vectors, each representing average speech recognition score for a SNR level. Then, the mean-centered matrix undergoes SVD to derive orthogonal latent variables (LVs) that capture significant covariance between HbO variability and behavioral score, highlighting optimal associations between brain variables and behavior variables. Typically, the number of experimental conditions or behavioral variables matches the number of LVs. Each LV comprises the singular value variable, which represents the strength of the effect that the LV represents. The singular vectors of brain saliences denote the brain variables most relevant to the behavior relationship.

Statistical testing occurs at two levels in behavioral PLS analyses. Initially, the overall significance of the LV is assessed through permutation tests. The significance of each LV pattern is assessed using 5,000 permutation tests. LVs are deemed significant if their singular value exceeds that of 95% of singular values derived from randomly shuffled data (permuted *p* < 0.05). Additionally, the reliability of each brain salience is evaluated using 5,000 bootstrap tests. A Bootstrap Ratio (BSR), normalized to assess robustness, is calculated by dividing the mean salience of each channel by its bootstrapped standard error. In this study, channels with bootstrap ratios larger than + 2 or smaller than − 2 were determined to be statistically significant corresponding to a 95% confidence level. Furthermore, bootstrap estimation is employed to determine 95% confidence intervals for each LV (Li et al., 2024). Non-overlapping confidence interval in behavioral PLS with zero suggests a significant correlation between HbO variability and behavioral performance.

### 2.8 Statistical analysis

We conducted a one-way ANOVA test to statistically analyze the demographic characteristics of three groups of participants. Significantly, the statistical analysis of gender adopted the two-sided chi-squared test. In addition, a mixed two-way ANOVA test (3 groups × 3 SNR conditions) was used to detect the impact of hearing loss groups and SNR conditions on behavioral performance (speech perception) and fNIRS signal variability. A detailed description of the statistics for each indicator has been described in the result section. SPSS 20.0 and Gretna statistical software were used in this study (Wang et al., 2015).

## 3 Results

### 3.1 Participants characteristics

We conducted one-way ANOVA and two-sided chi-squared test (only for gender) to examine the baseline level of demographic inclusion in three groups. Results showed that the groups were not different in age, gender, years of education (all *p* > 0.05). In addition, three groups of participants exhibited normal cognitive abilities (MoCA and MMSE) and depression status (GDS and UCLA), with no significant inter-group differences (Table 1, all *p* > 0.05). Remarkably, the one-way ANOVA test indicated substantial differences in hearing levels across the three groups [all F(2, 78) > 27.3, *p* < 0.001] at various frequency thresholds of the pure tone audiometry (0.25 kHz, 0.5 kHz, 1 kHz, 2 kHz, 4 kHz, and 8 kHz, Table 1). *Post hoc* comparisons revealed that the M_HI group exhibited significantly lower hearing levels compared to the HC group but better than the moderate to SM_HI group under different frequency threshold conditions.

**TABLE 1:** Demographic characteristics.

	HC, *n* = 30	M_HI, *n* = 25	SM_HI, *n* = 26	*P*-value
	**Mean (s.d.)**	**Mean (s.d.)**	**Mean (s.d.)**	
Age (years)	65.5 (3.4)	66.0 (3.7)	67.5 (3.7)	0.108^a^
Gender (male/famale)	14/16	12/13	13/13	0.585^b^
Education (years)	11.8 (1.6)	11.3 (2.2)	11.7 (2.0)	0.649^a^
MoCA	26.8 (1.8)	26.9 (1.9)	26.3 (2.8)	0.538^a^
MMSE	28.2 (1.5)	27.5 (1.8)	27.8 (1.5)	0.262^a^
GDS	5.3 (5.7)	4.2 (3.7)	5.0 (4.2)	0.663^a^
UCLA	34.2 (6.7)	33.0 (7.6)	34.6 (9.6)	0.748^a^
L_250	9.2 (4.5)	16.8 (8.9)	27.5 (15.4)	< 0.001^a^
L_500	10.5 (4.2)	20.4 (7.8)	35.2 (10.9)	< 0.001^a^
L_1000	15.0 (6.8)	24.6 (7.3)	42.5 (9.2)	< 0.001^a^
L_2000	12.7 (9.9)	29.6 (7.3)	48.7 (7.9)	< 0.001^a^
L_4000	21.0 (10.9)	39.6 (13.8)	57.3 (14.1)	< 0.001^a^
L_8000	27.5 (15.3)	49.0 (14.1)	63.1 (13.2)	< 0.001^a^
R_250	13.7 (5.9)	23.2 (9.0)	33.1 (13.5)	< 0.001^a^
R_500	12.5 (4.5)	22.2 (7.4)	36.2 (11.9)	< 0.001^a^
R_1000	11.7 (5.6)	16.8 (8.9)	25.0 (7.8)	< 0.001^a^
R_2000	13.3 (8.7)	30.4 (7.5)	50.6 (9.7)	< 0.001^a^
R_4000	20.0 (8.7)	41.2 (12.7)	57.3 (12.7)	< 0.001^a^
R_8000	25.8 (17.5)	48.8 (14.9)	61.5 (13.2)	< 0.001^a^

*^a^*One-way ANOVA.

*^b^*Two-sided chi-squared test.

### 3.2 Behavioral results

**FIGURE 2:**
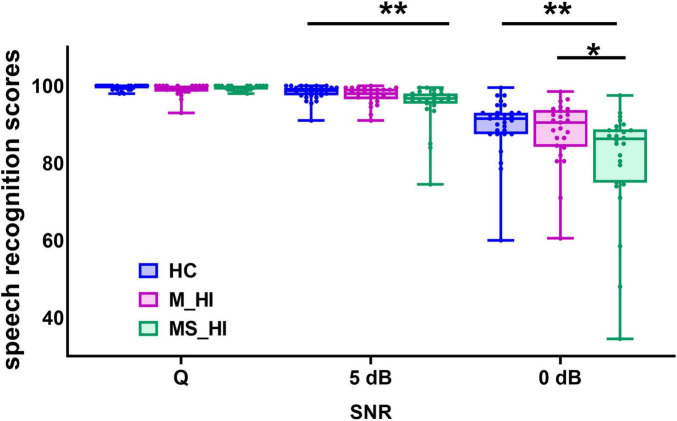
Comparison speech recognition scores among the three groups. Group means for speech recognition score under a quiet condition and 5 dB, 0 dB signal-to-noise ratio (SNR). The range of error bars represent the maximum and minimum values. HC, healthy control; M_HI, mild hearing loss; MS_HI, moderately severe hearing loss.; *p < 0.05; **p < 0.01.

The mixed two-way ANOVA test (3 groups × 3 SNR conditions) revealed that both the groups [F(2, 243) = 5.8, *p* = 0.005] and SNR conditions [F(2, 243) = 134.8, *p* < 0.001] significantly influenced the speech recognition scores (Figure 2). Additionally, there was a significant interaction between groups and SNR conditions [F(4, 243) = 5.9, *p* = 0.004). The simple effect analysis indicated significant differences in speech perception scores among the three groups at 5 dB SNR [F(2, 78) = 5.4, *p* = 0.006] and 0 dB SNR [F(2, 78) = 5.7, *p* = 0.005] noise conditions. For the condition of 5 dB SNR, *post hoc* comparisons showed that the MS_HI adults performed significantly worse in speech perception tasks than the HC adults (Bonferroni correction, *p* = 0.006), and a marginal statistical difference with M_HI adults (Bonferroni correction, *p* = 0.073). For the condition of 0 dB SNR, the speech perception scores for the MS_HI adults were also significantly lower than the HC adults (Bonferroni correction, *p* = 0.005) and the M_HI adults (Bonferroni correction, *p* = 0.049).

### 3.3 fNIRS signal variability results

**FIGURE 3:**
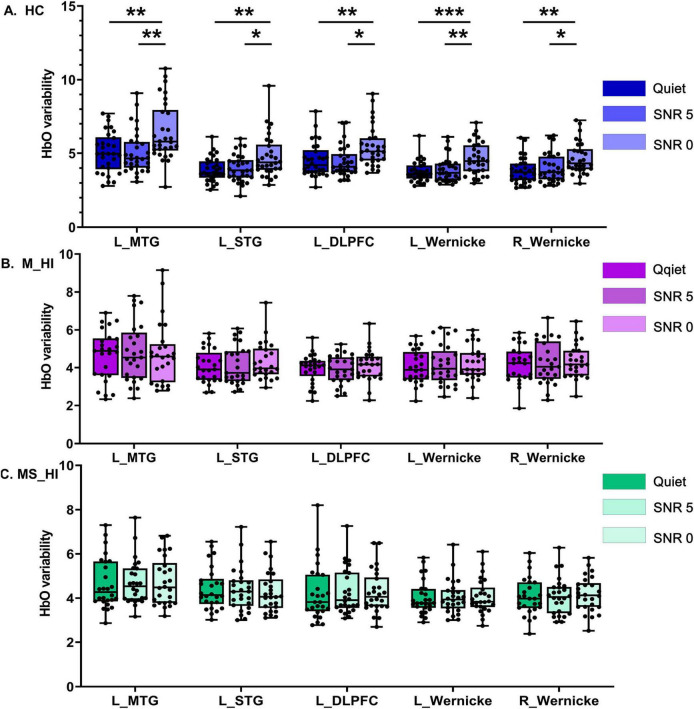
The main effect of SNR conditions. It is noteworthy that, due to the high variability of HbO in HC individuals, the range of the y-axis for this group is inconsistent with that of the other two groups. **(A)** Represents the HC group, **(B)** denotes the M_HI group, and **(C)** refers to the MS_HI group. HC, healthy control; M_HI, mild hearing loss; MS_HI, moderately severe hearing loss; MTG, middle temporal gyrus; SRG, superior temporal gyrus; DLPFC, dorsolateral prefrontal cortex; *p < 0.05; **p < 0.01; ***p < 0.001.

As described in the methodology section, we defined 12 symmetrical ROIs and conducted statistical analysis on the HbO variability recorded by the channels within these regions. Detailed coordinates are available in the Supplementary materials. A mixed two-way ANOVA test was employed to investigate the influence of hearing loss on HbO variability during speech recognition tasks under noisy conditions. The main effect of SNR conditions was found significant for the HbO variability in left MTG [*F*(2, 243) = 3.5, *p* = 0.04], left STG [*F*(2,243) = 3.8, *p* = 0.03], left DLPFC [*F*(2, 243) = 5.2, *p* = 0.008], left Wernicke’s area [*F*(2, 243) = 3.5, *p* = 0.04], and right Wernicke’s area [*F*(2, 243) = 3.6, *p* = 0.03]. We conducted a planned comparison to clarify the comparison between the SNR conditions under three groups. The results showed the HbO variability was significant SNR-related differences in HC group (Figure 3A) in left MTG [*F*(2, 87) = 7.5, *p* = 0.001], left STG [*F*(2, 87) = 6.1, *p* = 0.003], left DLPFC [*F*(2, 87) = 6.5, *p* = 0.002], left Wernicke’s area [*F*(2, 87) = 8.3, *p* = 0.001], and right Wernicke’s area [*F*(2, 87) = 6.3, *p* = 0.003]. *Post hoc* multiple comparison results revealed that HbO variability under low SNR condition (0 dB SNR) was significantly higher than that under high SNR condition (5 dB SNR) and quiet condition (Bonferroni correction, *P* < 0.05). Results indicated that higher levels of HbO variability associate with SNR load during the speech perception task. However, no association pattern between HbO variability and SNR loading was found in the other two hearing loss groups (Figures 3B, C).

**FIGURE 4:**
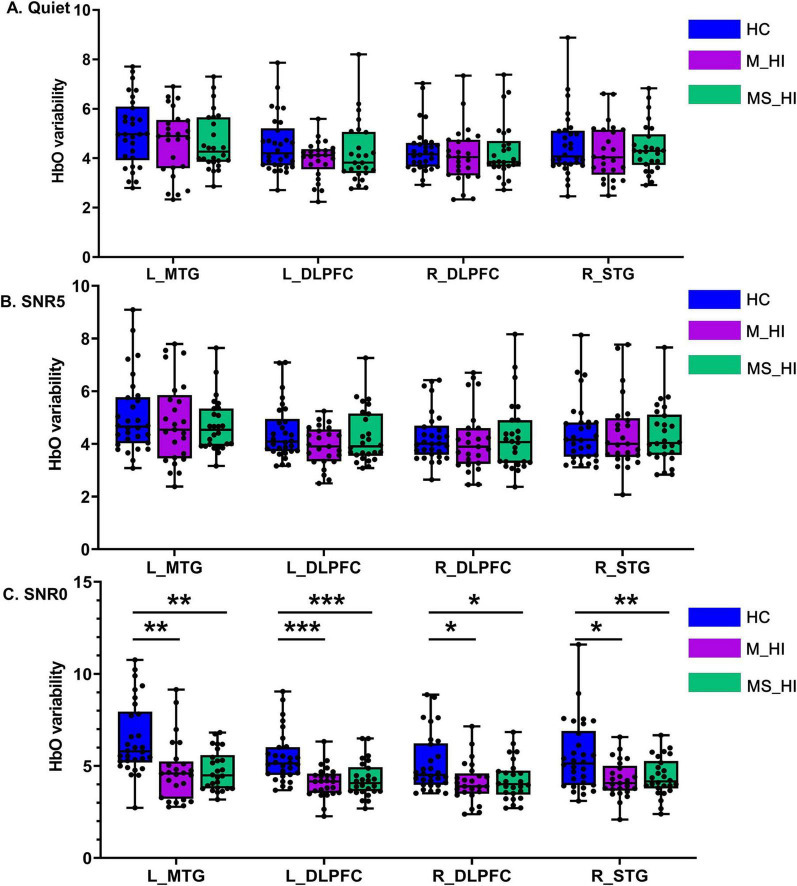
The main effect of group condition. **(A)** Represents the HC group, **(B)** denotes the M_HI group, and **(C)** refers to the MS_HI group. HC, normal hearing; M_HI, mild hearing loss; MS_HI, moderately severe hearing loss; MTG, middle temporal gyrus; SRG, superior temporal gyrus; DLPFC, dorsolateral prefrontal cortex; *p < 0.05; **p < 0.01; ***p < 0.001.

**FIGURE 5:**
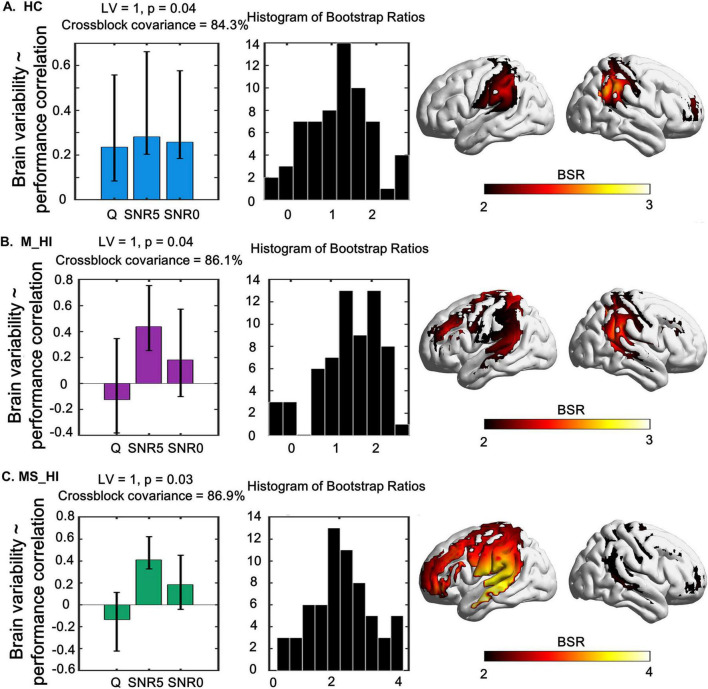
The relationship between task-modulated fNIRS signal variability and task-modulated performance using a behavior-PLS model are shown in left, and its associated spatial pattern expression in right. The middle column shows the distribution histogram of Bootstrap ratios. **(A)** Represents the HC group, **(B)** denotes the M_HI group, and **(C)** refers to the MS_HI group. HC, normal hearing; M_HI, mild hearing loss; MS_HI, moderately severe hearing loss. Yellow/red regions indicate greater up-modulation of brain variability with a decrease in accuracy. Error bars represent bootstrapped 95% confidence intervals. Bootstrap ratios increase from yellow to red, with a threshold value of greater than 2.00.

The main effect of group condition was also found significant for the HbO variability in left MTG [*F*(2, 243) = 25.6, *p* < 0.001], left DLPFC [*F*(2, 243) = 18.3, *p* < 0.001], right DLPFC [*F*(2, 243) = 8.9, *p* = 0.004], right STG [*F*(2, 243) = 12.3, *p* = 0.001]. Results of planned comparison showed HbO variability was significant group-related differences in the condition of 0 dB SNR (Figure 4C) in left MTG [*F*(2, 78) = 10.5, *p* < 0.001], left DLPFC [*F*(2, 78) = 12.3, *p* < 0.001], right DLPFC [*F*(2, 78) = 5.1, *p* = 0.008], right STG [*F*(2, 78) = 6.5, *p* = 0.020). *Post hoc* multiple comparison results revealed that HbO variability of HC group was greater than the M_HI and SM_HI group (Bonferroni correction, *P* < 0.05). However, no association pattern was found in the quiet (Figure 4A) and 5 dB SNR (Figure 4B) conditions. Results indicated that hearing loss could particularly affect the HbO variability during speech recognition tasks, especially in low SNR environments.

### 3.4 Behavioral PLS analysis: HbO variability and speech recognition performance

For each group, separate behavioral PLS analysis was conducted to correlate performance with changes in HbO variability under different conditions (i.e., quiet, 5 dB SNR, 0 dB SNR). We found a single significant LV that captured the relationship between HbO variability and speech recognition performance measures, and explained 84.3% of the crossblock covariance (permuted, *p* = 0.04) for the HC group. For the significant LV 1, greater HbO variability in bilateral STG, bilateral Wernicke’s area, and right DLPFC (Figure 5A) was associated with better speech recognition score during three conditions (i.e., quiet, 5 dB SNR, 0 dB SNR). We also found a single significant LV for the M_HI group (86.1% of the crossblock covariance, permuted, *p* = 0.04) and MS_HI group (86.9% of the crossblock covariance, permuted, *p* = 0.03). However, only the speech recognition score under the condition of 5 dB SNR was contribute to this LV. Specifically, greater HbO variability in bilateral STG, bilateral Wernicke’s area, left PMv, and left DLPFC was associated with better speech recognition score for the M_HI group (Figure 5B). And similar pattern was found in the M_HI group (Figure 5C).

## 4 Discussion

The main objective of this study was to utilize the HbO variability derived from fNIRS technique to explore how the brain signal variability of older adult changes in response to tasks of increasing SNR load and to examine the effects of varying degrees of hearing loss on speech recognition performance and related brain signal variability patterns. Results revealed that (1) brain signal variability increased with increasing SNR load in healthy older adults; (2) hearing loss influenced brain signal variability during speech recognition tasks, particularly under noisy conditions; (3) greater brain signal variability generally supported better speech recognition score in three SNR conditions (quiet, 5 dB SNR, 0 dB SNR) for the healthy older adults, but this pattern was only found under SNR5 conditions in the hearing loss groups.

Accumulating research has demonstrated that brain signal variability reveals the dynamic changes in brain states influenced by internal factors (Steinberg and King, 2024). Specifically, greater brain signal variability has been linked to healthy adults, with faster reaction time, more consistent performance and cognitive flexibility, in multiple tasks involving perception, perceptual matching, attentional, working memory, and cognitive cueing tasks (Clark et al., 2021; Garrett et al., 2011; Reinhart and Nguyen, 2019; Waschke et al., 2021). Our results showed that HbO variability in the left MTG, left STG, left DLPFC, and bilateral Wernicke’s area regions increased as the SNR load increased, showing an up-modulation of HbO variability in these regions in healthy older adults (Figure 3A). This load-dependent alteration in brain variability may signify the reinforcement of computational resources during the speech recognition tasks, and greater neural variability may indicate a more efficient neural system in individuals when performing multiple tasks. In fact, most previous studies have investigated how increased cognitive load in working memory tasks affects fNIRS signal variability (Li et al., 2024; Steinberg et al., 2022), but research on the impact of auditory cognitive load on brain signal variability has not yet been found. Our findings enhance and build upon prior research showing that brain variability is influenced by environmental factors and auditory task-related demands for the healthy older adults.

However, this similar significant variability was not found in the M_HI (Figure 3B) and SM_HI (Figure 3C) groups. In addition, we found that the HbO variability of M_HI and SM_HI groups in the left MTG, bilateral DLPFC, and right STG regions were significantly reduced compared to the HC group, particularly under extreme noisy conditions (0 dB SNR, Figure 4C). Numerous studies had shown that the overall brain signal variability across large-scale brain regions had emerged as a marker of a well-functioning brain (Guitart-Masip et al., 2016), and might be a potential biomarker for certain diseases, such as Alzheimer’s disease (Scarapicchia et al., 2019) and Parkinson’s disease (Maidan et al., 2022). Evidence also suggested that speech processing difficulties associated with hearing loss were not only associated with peripheral hearing loss but also with a cerebral decline across several functional networks (Harris et al., 2021; Zan et al., 2019), which might result in a neural system that was less flexible to speech recognition under noise environment. As introduced in the introduction, the variability of neural signals is fundamental for the flexible transition between high-integrated or segregated brain networks in metastable configurations. We speculate that hearing loss leads to neural dedifferentiation in auditory-related brain regions, resulting in a decrease in neural system flexibility in individuals with hearing loss. Therefore, our research demonstrated that hearing loss significantly reduced the modulation of HbO variability in response to auditory cognitive load tasks in older adults. We posited that future fNIRS research is necessary to explore the mechanisms and significance of HbO variability in the modulation related to age-related hearing loss and auditory cognitive processes.

Moreover, results of the PLS analysis that incorporated individuals’ speech recognition score by different SNR level demonstrated evidence for an interaction of SNR load and performance in the recruitment of the auditory circuitry-related regions. This multivariate approach showed that there was a significant positive correlation between brain signal variability and behavioral performance, that was, the higher HbO variability in bilateral STG, bilateral Wernicke’s area, and right DLPFC, the better speech recognition score in HC group (Figure 5A). Significantly, this association was found in all three SNR conditions, suggesting that the inter-individual brain variability-behavior relationship was stable and sensitive in HC older adults. Thus, we posited that brain signal variability could dynamically respond to the precise level of auditory environment demands. However, our results also showed that there was a significant positive correlation between brain signal variability and behavioral performance in M_HI group (Figure 5B) and MS_HI group (Figure 5C). And the brain regions with significant correlations increased, primarily including the left PMv, and left DLPFC, compared to the HC group, suggesting that hearing loss affected brain variability-behavior relationship. The source of changes in brain variability with hearing loss was still unclear (Maidan et al., 2022), but accumulating evidence suggested that altered regional variability may reflect sub-optimal functioning and compensatory mechanisms (Deco et al., 2009; Garrett et al., 2013). Evidence also suggested that the increased activity in the frontal cortex (Du et al., 2016) and PMv (Wong et al., 2009) compensated for impaired speech perception in age related high-frequency hearing loss older adults. Overall, our findings were consistent with previous studies that the recruitment of the left PMv, and left DLPFC might provide a means of compensation in older adults with hearing loss for decoding speech in the adverse listening environment.

Of note, the association of brain variability-behavior relationship was only evident in the 5 dB SNR condition, suggesting that this relationship might be affected by SNR load in hearing loss adults. Specifically, the speech recognition scores were relatively high and concentrated in quiet condition compared to noise conditions (Figure 2), and the sensitivity of brain variability to hearing loss decreased (Figure 4A). These findings may suggest the presence of a ceiling effect, whereby participants have achieved their maximal performance levels. This phenomenon could elucidate the lack of significant correlations between variability and performance under these conditions. In addition, the non-significant result was also found for the 0 dB SNR condition. Researchers have argued that if a task is too difficult, people may disengage from it or simply “give up,” since it exceeds one’s capability (Causse et al., 2017; Mandrick et al., 2013). And participants may have varying thresholds for noise tolerance, which could influence their performance in challenging listening environments. We infer that the 0 dB SNR condition might not be reflecting the highest auditory cognitive load across all participants. Results of a multivariate analysis including performance and standard task-evoked activation further support this idea (Meidenbauer et al., 2021). Thus, the large individual differences and generally poor performance of the 0 dB SNR condition may explain the non-significant results in this study and should be examined in more detail in future work.

There are several issues that may limit the interpretability of our findings that should be mentioned. First, although our results suggest that hearing loss is associated with a decrease in the variability of brain signals. However, no significant differences were observed between the M_HI group and MS_HI group. These might be due to the overlap in auditory characteristics between mild and the moderate to severe hearing loss. The sensitivity of sample size may also affect the results, leading to similar performance between the two groups. Future studies should consider increasing the sample size of patients with varying degrees of hearing loss to further elucidate the effects of hearing loss severity on the variability of brain signals. Additionally, this study included only three different SNR levels, and the association of brain variability-behavior relationship was only evident in the 5 dB SNR condition. Future studies could set more levels in order to gain further insight into the relationship between brain signal variability and behavioral performance.

In conclusion, this study utilized fNIRS-derived HbO variability to investigate how brain signal variability in older adults responds to tasks of increasing SNR load and examined the impact of varying degrees of hearing loss on speech recognition performance and related brain signal variability patterns. Our study highlights the significant impact of hearing loss on brain signal variability modulation in auditory cognitive tasks and underscores the need for further research in this area. In addition, the insights derived from the variability-performance correlations may inform the design of assessments aimed at measuring auditory cognitive processing capabilities. This might enhance the ability to assess individual differences and tailor interventions to meet specific auditory processing needs.

## Data Availability

The datasets generated and/or analysed during the current study are not publicly available to protect participant’s confidentiality but are available from the corresponding author upon reasonable request. Requests to access these datasets should be directed to humwang2016@163.com.
