# Supplementary material for: Impact of hearing loss on brain signal variability in older adults under different auditory load conditions
Source: Front Aging Neurosci. 2025 Feb 27;17:1498666. doi: 10.3389/fnagi.2025.1498666 (PMC11903437; doi:10.3389/fnagi.2025.1498666)
Supplement: Supplementary file 1 [file Table_1.docx]

**Supplementary materials**


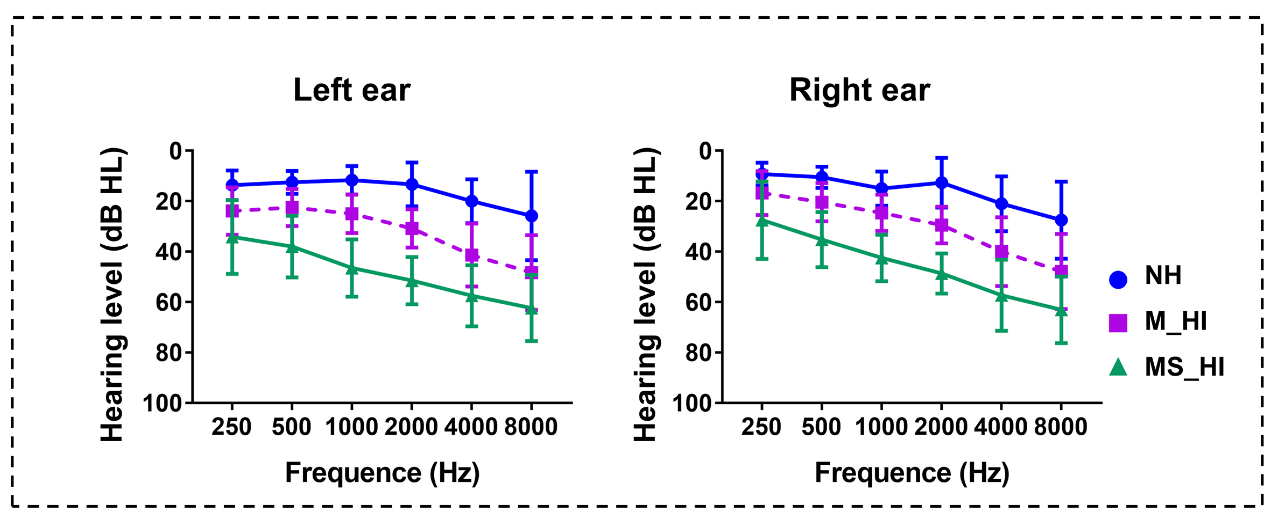


Figure S1. Comparison of hearing thresholds among the three groups. Group means for pure-tone hearing thresholds at each frequency. Error bars indicate standard deviation (SD)

Table S1 MNI coordinates corresponding to the channel location of ROI.

| Regions of interest | Channel（BA） | Hemisphere | MNI | | | Percentage of overlap |
| --- | --- | --- | --- | --- | --- | --- |
|  |  |  | x | y | z |  |
| Superior Temporal Gyrus | 6（22） | Left | -70 | -20 | 11 | 0.83 |
|  | 57（22） | Right | 69 | -20 | 15 | 0.83 |
| Middle Temporal gyrus | 1（21） | Left | -62 | 4 | 31 | 0.81 |
|  | 58（21） | Right | 68 | -8 | -5 | 0.73 |
| Broca's area | 11（45） | Left | -54 | 34 | 27 | 0.98 |
|  | 51（45） | Right | 53 | 35 | 26 | 1 |
| Wernicke's area | 16（40） | Left | -63 | -51 | 42 | 0.82 |
|  | 53（40） | Right | 63 | -46 | 47 | 0.99 |
| dorsolateral prefrontal cortex | 9（47） | Left | -53 | 45 | -4 | 0.63 |
|  | 50（47） | Right | 53 | 45 | -5 | 0.61 |
| Ventral premotor cortex | 27（6） | Left | -23 | -8 | 76 | 1 |
|  | 45（6） | Right | 23 | -6 | 76 | 1 |
